# Candidemia and invasive candidiasis in children and neonates: transposing adult diagnostic recommendations may undermine end-organ screening

**DOI:** 10.1016/j.bjid.2026.105966

**Published:** 2026-09-17

**Authors:** Fabrício Silva Pessoa

**Affiliations:** Hospital Universitário da Universidade Federal do Maranhão (UFMA), Pediatric Infectious Diseases Department, São Luis, MA, Brazil

Dear Editor,

We read with great interest the recent “Brazilian guidelines for the diagnosis and treatment of candidemia and invasive candidiasis in adults and children” by Colombo et al.^1^ By consolidating national epidemiological data and adapting the best international evidence to the Brazilian healthcare setting, this document makes a substantial and long-awaited contribution that is likely to become the main practical reference for candidemia management in the country. Precisely because of this anticipated impact on bedside practice, we would like to raise three points concerning the pediatric and neonatal population, whose consideration could further strengthen the guidelines.

First, the statement that no pediatric case of *Candida auris* has been reported in Brazil is outdated. The guidelines note that, although *C. auris* infections have been documented in children and neonates in Venezuela and Colombia, no pediatric case has been reported in Brazil.^1^ This does not reflect national surveillance data. The Brazilian Health Regulatory Agency (Anvisa) Risk Alert GVIMS/GGTES n° 01/2023 documented *C. auris* in a preterm neonate admitted to a neonatal intensive care unit in São Paulo ‒ first in a rectal surveillance swab and subsequently in blood and urine cultures, that is, with invasive disease ‒ representing the 77th case in the country and the first in the state of São Paulo.^2^ This is therefore not merely a pediatric case but a neonatal bloodstream infection. Moreover, the assertion sits in tension with the guidelines’ own epidemiological narrative, which elsewhere lists São Paulo among the states with reported *C. auris* outbreaks ‒ the very state in which this neonatal case occurred ‒ so the discrepancy is internal to the document, independent of the external Anvisa source. The correction matters because the perception that Brazilian children are spared from *C. auris* may discourage active surveillance and screening in at-risk neonates and infants ‒ the very group in which colonization and nosocomial transmission of this pathogen are hardest to contain.

Second, and with the greatest clinical impact, the recommendations for end-organ screening were built on adult evidence and transposed to children without adaptation. In line with current adult practice, the guidelines abandon universal fundoscopy and echocardiography in favor of a selective, symptom- and risk-based approach.^1^ For adults this shift is defensible: the incidence of endophthalmitis and endocarditis is low, and patients can usually report visual complaints or display signs ‒ a new murmur, embolic phenomena ‒ that trigger investigation. In the preverbal child and, above all, in the neonate, this logic is inverted. The signs and symptoms of candidemia and its metastatic complications are nonspecific and subtle, and the visual complaint ‒ the central trigger of the adult algorithm ‒ is, by definition, inaccessible. At the same time, hematogenous dissemination to end organs is more frequent: hematogenous *Candida* meningoencephalitis is favored by the greater blood–brain barrier permeability of the preterm infant, and central nervous system involvement is substantially more common than in adults. For these reasons, the IDSA update.^4^ recommends, systematically rather than selectively, a dilated retinal examination and lumbar puncture in every neonate with a positive blood or urine culture for *Candida*, as a strong recommendation. The ECMM/ISHAM/ASM global guideline cited by the authors themselves.^3^ likewise calls, in every case of pediatric candidemia, for clinical evaluation of deep sites of infection including an ophthalmologic examination ‒ though, tellingly, only within its treatment chapter and without assigning a formal strength-of-recommendation grade, thereby reproducing at the international level the same subordination we identify in the Brazilian document.

The pediatric section of the guidelines does state that all pediatric patients with candidemia should undergo a thorough search for deep-organ involvement, including ocular and central nervous system evaluation.^1^ However, this guidance appears in a subordinate, generic form, overshadowed by the document’s dominant architecture, which devotes dedicated sections to the selective indication of fundoscopy and echocardiography, both built on adult premises. The practical risk is that a reader ‒ faced with an adult chapter declaring fundoscopy “no longer universally recommended” ‒ will carry that conduct over to the pediatric patient, underdiagnosing chorioretinitis and endophthalmitis precisely where they cannot be clinically suspected.

Third, echocardiography lacks a dedicated pediatric recommendation. The risk factors listed to indicate it ‒ a new murmur, prosthetic valves or cardiac implantable electronic devices, chronic hemodialysis for more than three months, injection drug use, and persistent candidemia^1^ ‒ are almost entirely adult criteria, inapplicable to the neonate. Neonatal *Candida* endocarditis presents differently: it typically occurs in preterm infants with persistent candidemia associated with a central venous catheter, frequently affects structurally normal hearts, and may run without an audible murmur, silently or with late onset.^3^^,^^5^ Transposing adult triggers means, in practice, failing to order echocardiography in exactly the population in which clinical suspicion is least reliable. Accordingly, assessment for disseminated disease in the neonate should routinely include ‒ beyond fundoscopy and lumbar puncture ‒ echocardiography and cranial and abdominal ultrasonography.^3^^,^^4^

We respectfully suggest that a future revision of the guidelines (i) Correct the statement on the absence of pediatric *C. auris* in Brazil in light of Anvisa Alert n° 01/2023.^2^ and (ii) Explicitly decouple the pediatric and neonatal diagnostic recommendations from the adult selective paradigm, establishing, for every child ‒ and especially every neonate ‒ with candidemia or candiduria, systematic end-organ screening: dilated fundoscopy, echocardiography, lumbar puncture, and neuroimaging. In a population in which the clinical examination is intrinsically limited, systematic screening is not diagnostic excess; it is the only way to avoid missing manifestations that substantially change the duration and choice of antifungal therapy.

We congratulate the authors on this timely and important document and hope these comments contribute to its continued improvement.

## Authors’ contributions

Fabrício Silva Pessoa: Conceived the letter; Performed the literature review; drafted and critically revised the manuscript, and approved the final version.

## Conflicts of interest

The authors declare no conflicts of interest.

## Data Availability

All data discussed are available within the cited publications and public documents; no new data were generated for this letter.
